# Unintended femoral valgization after distal femoral derotational osteotomy in a patient with recurrent patella dislocation: A case report

**DOI:** 10.1097/MD.0000000000046339

**Published:** 2025-12-12

**Authors:** Hyunkwon Kim, Jisu Park, Tae Woo Kim, Moon Jong Chang

**Affiliations:** a Department of Orthopaedic Surgery, SMG-SNU Boramae Medical Center, Seoul, Republic of Korea; b Department of Orthopaedic Surgery, Seoul National University College of Medicine, Seoul, Republic of Korea.

**Keywords:** distal femoral derotational osteotomy, femoral anteversion, patellar dislocation, valgus alignment

## Abstract

**Rationale:**

Distal femoral derotational osteotomy (DFDO) is widely performed to correct excessive femoral anteversion in patients with patellofemoral instability. However, unintended changes in coronal alignment can occur depending on the orientation of the cutting plane. We present a case of unintentional femoral valgization after DFDO in a patient with a recurrent patellar dislocation.

**Patient concerns:**

A 23-year-old female presented with multiple episodes of recurrent patellar dislocation of her right knee.

**Diagnoses:**

Physical examination revealed a positive apprehension test, positive J-sign, and generalized joint laxity. An effusion was observed in the right knee. Radiologic findings included increased femoral anteversion (30.3°), ruptured medial patellofemoral ligament (MPFL), mild varus alignment (hip-knee-ankle angle 0.4°), mechanical lateral distal femur angle (mLDFA) of 86.6°, patella alta, and increased tibial tuberosity–trochlear groove (TT–TG) distance.

**Interventions:**

The patient underwent MPFL reconstruction and DFDO with target correction of femoral anteversion of 15°. Osteotomy was performed perpendicular to the real anatomical shaft axis.

**Outcomes:**

Postoperative imaging revealed an unintended valgus malalignment (HKA angle −3.0°) and decrease of mLDFA (83.6°). Despite the altered alignment, no recurrence of dislocation was noted during the 18-month follow-up period, and the clinical outcome was satisfactory.

**Lessons:**

Unintentional valgus alignment may result from osteotomies oriented perpendicular to the real anatomical shaft axis. Surgeons should consider using the virtual anatomical shaft axis to determine the cutting plane and minimize the risk of iatrogenic malalignment during DFDO.

## 1. Introduction

Distal femoral derotational osteotomy (DFDO) is typically recommended in patients with symptoms and excessively increased femoral anteversion. This surgery is recommended for patients with symptomatic malalignment of the patellofemoral joint and femoral anteversion >25°–30°. The aim of surgery is to reduce anteversion to a normal range (15°) by externally rotating the distal fragment, thereby correcting the transverse alignment of the knee.^[1]^

While correcting the specific cause that necessitates surgery is crucial, care must also be taken to ensure that other risk factors do not worsen. As the risk factors are not uniform across all patients, an individualized approach is necessary, focusing on treating the underlying key pathoanatomy. The pathophysiology of recurrent patellar dislocation is multifactorial, involving factors such as trochlear dysplasia, increased Q-angle, increased femoral anteversion, soft tissue contracture, and patella alta.

In this report, we present a case of unintended valgization following DFDO in a patient with recurrent patellar dislocation.

## 2. Case report

A 23-year-old female presented with a history of recurrent patellar dislocation of her right knee. The initial dislocation occurred 7 months prior to the fall and was treated conservatively with a 6-week long leg cast. She remained asymptomatic until 1 month prior to presentation, when pivoting resulted in another dislocation, followed by 5 to 6 further episodes within 3 days.

Physical examination revealed a positive apprehension test, positive J-sign, and generalized joint laxity. An effusion was observed in the right knee.

**Figure 1.**
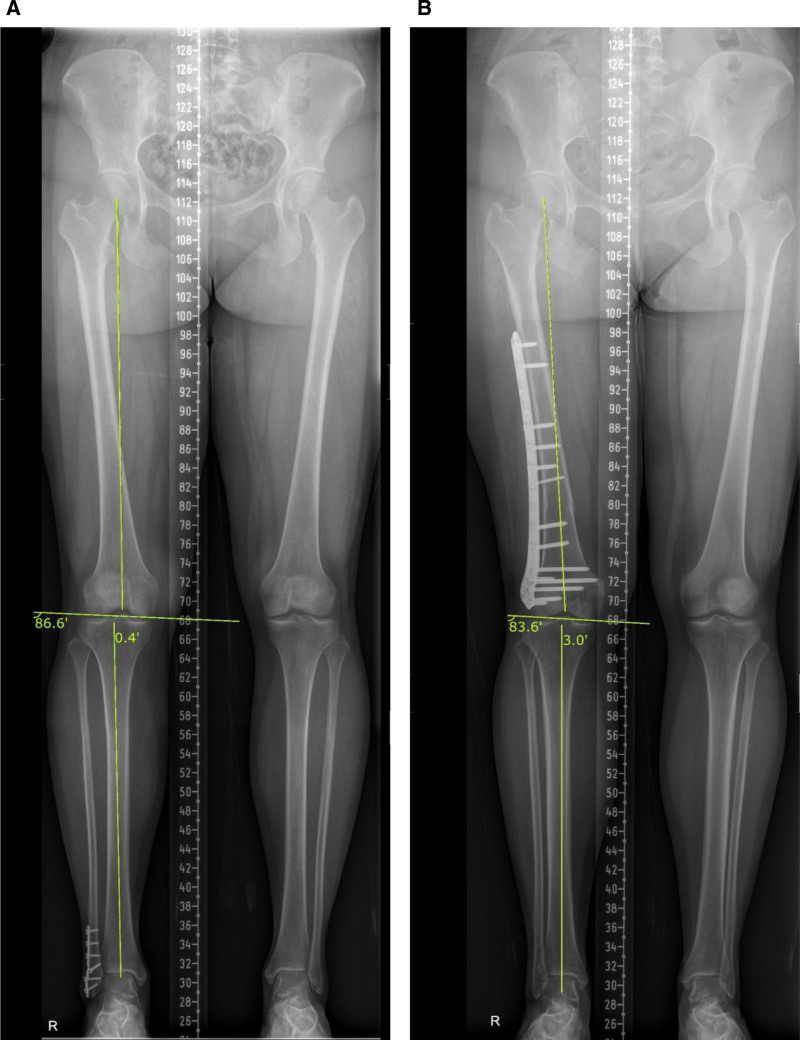
(A) Preoperative lower leg alignment. Varus. HKA 0.4°. mLDFA 86.6°. (B) Postoperative lower leg alignment. Valgus. HKA –3.0°, mLDFA 83.6° HKA = hip-knee-ankle, mLDFA = mechanical lateral distal femur angle.

In the radiological examination, only an increase in femoral anteversion and ruptured medial patellofemoral ligament (MPFL) were definite indications for surgery, while other factors were borderline. Radiographic imaging demonstrated patella alta (Insall-Salvati ratio: 1.28), a mild varus hip-knee-ankle (HKA) angle (0.4°) and mechanical lateral distal femur angle (mLDFA) of 86.6° (Fig. 1A). Magnetic resonance imaging revealed complete rupture of the MPFL with no evidence of loose bodies. Computed tomography (CT) revealed a tibial tuberosity–trochlear groove (TT–TG) distance of 17 mm and femoral anteversion of 30.3°.

MPFL reconstruction and DFDO were performed given the MPFL rupture and excessive femoral anteversion. Tibial tubercle transfer was not performed because the TT–TG distance (17 mm) was within the borderline range, and no excessive lateralization or trochlear dysplasia was observed. Therefore, correction of femoral anteversion with DFDO combined with MPFL reconstruction was considered sufficient to restore patellofemoral tracking. The planned correction angle was set at 15° to achieve femoral anteversion of 15°, which represents the upper limit of the normal range. Before performing the osteotomy, 2 Schanz pins were inserted into the anterior cortex of the proximal and distal fragments to monitor the rotational angle. Osteotomy was performed perpendicular to the distal femoral shaft axis at the surgical window. After completing the osteotomy, the distal fragment was externally rotated until the 2 pins formed an angle of 15°, corresponding to the planned derotation. The procedure was performed under C-arm fluoroscopic guidance, and fixation was achieved with a lateral distal femoral locking plate (Tomofix; Synthes, Oberdorf, Switzerland). No computer navigation system was used.

Postoperative teleradiograph revealed unexpected valgus alignment of the HKA angle (−3°) and decrease of mLDFA (83.6°) (Fig. 1B). This was an unexpected finding in the surgical plan and a potential risk factor for recurrent patellar dislocation after surgery. However, at 1 year and 6 months post-surgery, there was no recurrence of patellar dislocation during outpatient follow-up, and the result was satisfactory with a KSS score of 58.6 points and a Kujala score of 80 points.

## 3. Discussion

DFDO is a widely accepted surgical procedure for addressing increased femoral anteversion in patients with patellofemoral instability. However, as shown in the present case, such procedures may lead to unintended valgus malalignment, which paradoxically represents a recognized independent risk factor for recurrent patellofemoral instability.^[2–5]^ Understanding the biomechanical mechanisms and surgical considerations underlying this complication is therefore of clinical importance.

Previous reports have indicated that external derotational osteotomies at the distal femur may alter coronal plane alignment. Nelitz et al demonstrated using a computer model that distal external derotational osteotomies often result in valgization, whereas proximal osteotomies tend to induce varization.^[6]^ Similarly, Konrads et al reported in a retrospective series that supracondylar external rotation osteotomies cause valgization of the coronal limb alignment and enlargement of the ischiofemoral space.^[7]^ These findings are consistent with our case, in which an unexpected valgus shift of the HKA angle occurred following DFDO.

**Figure 2.**
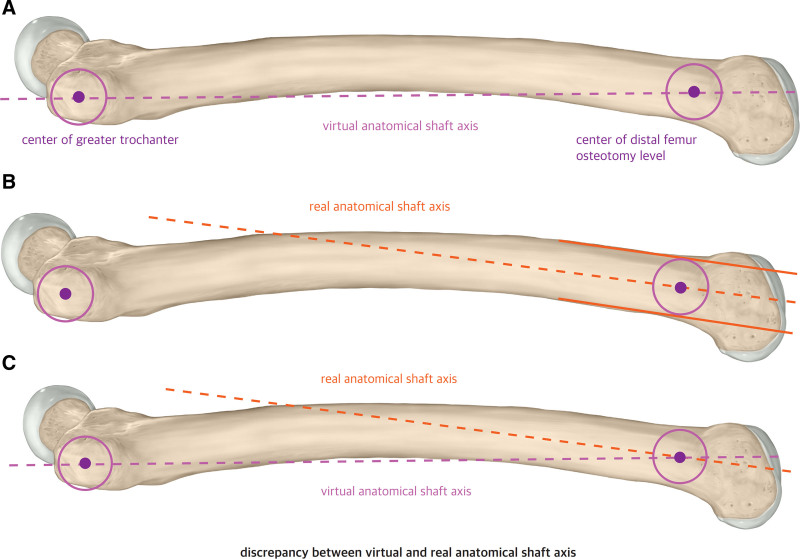
(A) Virtual anatomical shaft axis. Line extending from the center of the palpated greater trochanter to the center of the desired osteotomy site in the lateral view. (B) Real anatomical shaft axis. Line parallel to femur shaft at the level of osteotomy site. (C) The discrepancy between the virtual and real anatomical shaft axes.

**Figure 3.**
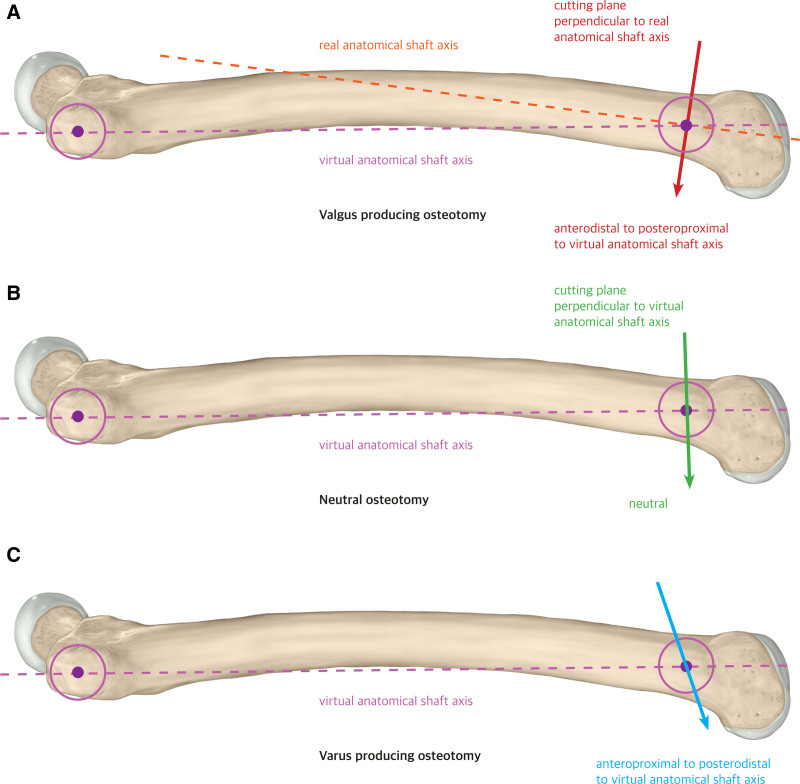
(A) Valgus-producing osteotomy. Cutting plane perpendicular to the real anatomical shaft axis. Cutting plane perpendicular to the femoral shaft from the surgical window. Cutting plane anterodistal to posteroproximal to the virtual anatomical shaft axis. (B) Neutral osteotomy. Cutting plane perpendicular to the virtual anatomical shaft axis. (C) Varus-producing osteotomy. Cutting plane anteroproximal to posterodistal to the virtual anatomical shaft axis.

More recently, Imhoff et al proposed that unintended valgization is strongly influenced by the orientation of the cutting plane relative to the femoral shaft with their “Pillar-Crane” concept study.^[8]^ They defined the “virtual anatomical shaft axis” as a line extending from the center of the palpated greater trochanter to the center of the desired osteotomy site in the lateral view (Fig. 2A). When osteotomies performed perpendicular to the virtual anatomical shaft axis, the mLDFA slightly increases, which helps prevent unintended valgus malalignment (Fig. 3B). Conversely, if osteotomy is performed perpendicular to the real anatomical shaft axis (Fig. 2B), as is frequently done in clinical practice, the cutting plane is oriented from anterodistal to posteroproximal relative to the virtual anatomical shaft axis, leading to a reduction in the mLDFA and potential valgization of the femur (Fig. 3A). Furthermore, when the cutting plane is directed anteroproximal to posterodistal relative to the virtual anatomical shaft axis, femoral varization occurs (Fig. 3C), which may be beneficial in patients with recurrent patellar dislocation, where valgus alignment is a risk factor. A subsequent cadaveric study confirmed that an oblique single-cut osteotomy, based on preoperatively defined angles, can simultaneously correct torsion and frontal alignment within a clinically negligible margin of error.^[9]^

Previous studies have reported unintended valgization following DFDO,^[6]^ which has been attributed to the frequent use of a cutting plane perpendicular to the real anatomical shaft. This is mainly because surgeons tend to perform osteotomy perpendicular to the femoral shaft with limited sight from the surgical window. In patients with excessive femoral anteversion, the discrepancy between the virtual and real anatomical shaft axes (Fig. 2C) tends to be more pronounced due to femoral bowing, which positions the greater trochanter further posteriorly relative to the distal femur center, an effect that becomes more evident as anteversion increases.

**Figure 4.**
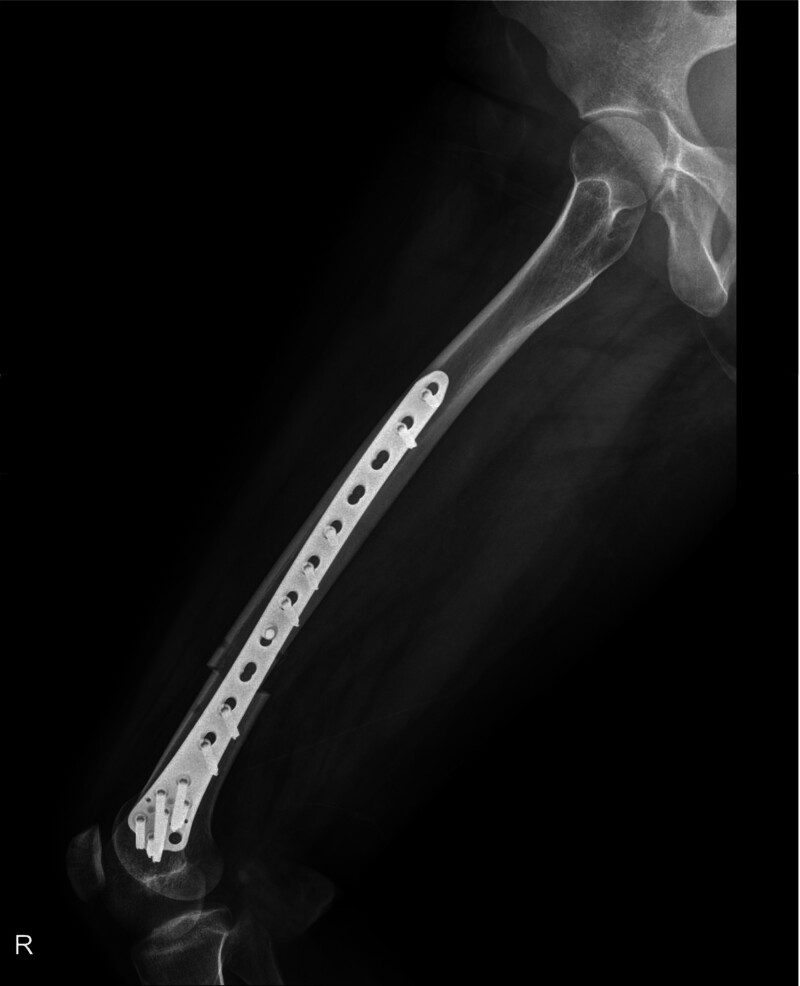
Postoperative radiograph. The osteotomy level was slightly proximal due to the contour of the distal femur and the length of the fixation plate. The small anterior cortical holes correspond to the Schanz pin tracks used intraoperatively to confirm 15° derotation. The cutting plane was oriented from anterodistal to posteroproximal, resulting in unintended femoral valgization.

In the present case, the osteotomy was performed perpendicular to the real anatomical shaft axis, and the cutting plane was oriented from anterodistal to posteroproximal, likely contributing to unintended femoral valgization (Fig. 4). This aligns with Imhoff biomechanical framework and underscores the importance of referencing the virtual shaft axis when planning and executing distal femoral derotational osteotomies.

The clinical relevance of this case lies in the potential risk of recurrent instability when valgus alignment is introduced after derotational osteotomy. Although our patient remained free of redislocation during follow-up, valgus malalignment is an established risk factor for patellofemoral instability and may also contribute to unfavorable long-term outcomes. To minimize the risk of unintentional valgus malalignment, it is recommended to reference the virtual anatomical shaft axis when planning osteotomy and orienting the cutting plane from anteroproximal to posterodistal. Further research is necessary to quantify the precise impact of the cutting plane orientation on lower limb alignment.

This report has several limitations. First postoperative CT was not obtained due to radiation concerns and the absence of clinical indication. Although CT-based assessment would have provided more precise quantification of the torsional correction, it was omitted to minimize radiation exposure. Instead, long-standing radiographs were used to evaluate postoperative alignment and were deemed sufficient for clinical follow-up. Second, as a single case report, the findings cannot be generalized to all patients undergoing DFDO. Despite these limitations, this case highlights an important potential complication of DFDO and provides biomechanical insight for surgical planning.

## Acknowledgments

The authors thank the patient for her cooperation.

## Author contributions

**Conceptualization:** Hyunkwon Kim, Moon Jong Chang.

**Supervision:** Moon Jong Chang.

**Validation:** Jisu Park, Tae Woo Kim, Moon Jong Chang.

**Visualization:** Hyunkwon Kim.

**Writing – original draft:** Hyunkwon Kim.

**Writing – review & editing:** Hyunkwon Kim, Jisu Park, Tae Woo Kim, Moon Jong Chang.
